# Reductive dissolution of pyrite by methanogenic archaea

**DOI:** 10.1038/s41396-021-01028-3

**Published:** 2021-06-10

**Authors:** Devon Payne, Rachel L. Spietz, Eric S. Boyd

**Affiliations:** Department of Microbiology and Immunology, Montana State University, Bozeman, MT USA

**Keywords:** Biogeochemistry, Water microbiology

## Abstract

The formation and fate of pyrite (FeS_2_) modulates global iron, sulfur, carbon, and oxygen biogeochemical cycles and has done so since early in Earth’s geological history. A longstanding paradigm is that FeS_2_ is stable at low temperature and is unavailable to microorganisms in the absence of oxygen and oxidative weathering. Here, we show that methanogens can catalyze the reductive dissolution of FeS_2_ at low temperature (≤38 °C) and utilize dissolution products to meet cellular iron and sulfur demands associated with the biosynthesis of simple and complex co-factors. Direct access to FeS_2_ is required to catalyze its reduction and/or to assimilate iron monosulfide that likely forms through coupled reductive dissolution and precipitation, consistent with close associations observed between cells and FeS_2_. These findings demonstrate that FeS_2_ is bioavailable to anaerobic methanogens and can be mobilized in low temperature anoxic environments. Given that methanogens evolved at least 3.46 Gya, these data indicate that the microbial contribution to the iron and sulfur cycles in ancient and contemporary anoxic environments may be more complex and robust than previously recognized, with impacts on the sources and sinks of iron and sulfur and other bio-essential and thiophilic elements such as nickel and cobalt.

## Introduction

Pyrite (FeS_2_) is the most abundant sulfide mineral in Earth’s crust and its formation and fate are key controls on the biogeochemical cycles of iron (Fe), sulfur (S), carbon, and oxygen (O_2_) [1]. However, the specific role of sulfide minerals, such as FeS_2_, in these cycles has changed drastically over geological time [2, 3]. Isotopic evidence suggests that FeS_2_ has formed in sedimentary or volcanic environments since at least 3.81 Gya [4]. Yet, prior to the advent of oxygenic photosynthesis and the gradual accumulation of O_2_ ~2.4 Gya (as reviewed in [5, 6]), biotic and abiotic oxidative weathering of FeS_2_ with O_2_, the primary driver of sulfate (SO_4_^2−^) input into oceans [7], was of minimal importance [3, 6, 8, 9]. Furthermore, reduction of FeS_2_ has only been demonstrated at high temperature (>90 °C) in abiotic laboratory reactors containing artificially high (>15 bar) concentrations of hydrogen (H_2_) [10, 11]. Together, these data imply that once iron sulfide minerals such as FeS_2_ form in low temperature sulfidic and anoxic environments, they remain largely unavailable to biology since they cannot be extensively mobilized through oxidative or reductive weathering [3]. This raises key questions as to how anaerobic microorganisms acquire Fe (and other thiophilic metals such as nickel and cobalt) and S to meet biosynthetic demands in contemporary and past anoxic environments that favor formation of iron sulfide minerals, such as sulfidic freshwater or marine sediments [1, 12–14].

All cells require Fe and S, which are key components of amino acids, vitamins, and a variety of co-enzymes and co-factors. This includes both simple and complex biological iron–sulfur ([Fe-S]) clusters that function in electron transfer, catalysis, substrate binding and activation, and other purposes [15, 16]. As a consequence of their functional versatility, [Fe-S] clusters are ubiquitous in all life forms [15, 17] and have central roles in photosynthesis, respiration, and fermentation [18].

Methanogenic archaea are argued to be the most primitive of extant organisms [19, 20], and isotopic evidence preserved in the rock record indicates their presence as early as ~3.46 Gya [21]. Despite their early emergence during the anoxic Archean, when free Fe and/or S may have been limited due to precipitation as sulfide minerals [2, 3], methanogens have been shown to utilize [Fe-S] clusters more extensively than other organisms. Specifically, *Methanococcus maripaludis* cells were shown to contain ~15 fold more [Fe-S] clusters per mg protein than *Escherichia coli* cells [22] and methanogen genomes code for a higher number of putative [Fe-S] cluster binding proteins than those of facultative anaerobes and obligate aerobes [23].

The types of S sources that support methanogens vary based on their evolutionary history. Methanogens that belong to more primitive (Class I) lineages (Methanopyrales, Methanobacteriales, and Methanococcales) tend to be grown with sulfide (HS^−^/H_2_S) as their S source whereas more recently evolved (Class II) lineages (Methanocellales, Methanomicrobiales, and Methanosarcinales) can also be grown with cysteine as their sulfur source [22, 24]. Sulfide is toxic to methanogens [25], but toxicity can be alleviated by precipitation with metals, such as ferrous iron (Fe(II)), nickel (II), or cobalt (II) [26]. Sulfide readily reacts with Fe(II), resulting in the formation of transient aqueous iron monosulfide clusters (FeS_(aq)_) via Eq. (1) [27] that can condense to form metastable and less soluble nanoparticulate iron sulfide mineral phases such as mackinawite (FeS_(mack)_) via Eq. (2) [27–29]. Depending on the type and abundance of aqueous S species in an environment (e.g., H_2_S or polysulfide (S_x_^2−^)), dissolution and/or solid-state transformation of FeS_(mack)_ can occur and can result in the formation of FeS_2_ via Eqs. (3) and (4) [13, 27, 29]:

1 $$H_2{\mathrm{S}}_{{\mathrm{(aq)}}} + {\mathrm{Fe}}\left( {{\mathrm{II}}} \right)_{{\mathrm{(aq)}}} \to {\mathrm{FeS}}_{{\mathrm{(aq)}}} + {\mathrm{2H}}^{\mathrm{ + }}$$

2 $${\mathrm{FeS}}_{{\mathrm{(aq)}}} \to {\mathrm{FeS}}_{{\mathrm{(mack)}}}$$

3 $${\mathrm{FeS}}_{{\mathrm{(mack)}}} + {\mathrm{H}}_{\mathrm{2}}{\mathrm{S}}_{{\mathrm{(aq)}}} \to {\mathrm{FeS}}_{\mathrm{2}} + {\mathrm{H}}_{{\mathrm{2(g)}}}$$

4 $${\mathrm{FeS}}_{({\mathrm{mack}})} + {\mathrm{S}}_{\mathrm{X}}^{2 - }\left( {{\mathrm{aq}}} \right) \to {\mathrm{FeS}}_{\mathrm{2}} + {\mathrm{S}}_{{\mathrm{x}} - {\mathrm{1}}}^{2 - }\left( {{\mathrm{aq}}} \right)$$

The anoxic, sulfidic environments that anaerobic methanogens inhabit can have high concentrations of Fe(II), which would tend to favor the formation of iron sulfide minerals including FeS_(mack)_ and FeS_2_ [27, 29]. However, FeS_2_ is reportedly stable under anoxic, low temperature conditions [10, 11] and, to date, has not been shown to be bioavailable to microorganisms in the absence of oxygen or alternative oxidants (e.g., manganese oxides, ferric iron ions) that themselves require O_2_ to generate [30].

Here, we characterized the growth and methanogenesis activity of the model Class I and II methanogens, *Methanococcus voltae* strain A3 and *Methanosarcina barkeri* strain MS, respectively, in defined medium (Supplementary Online Materials (SOM) Tables S1 and S2). *M. voltae* A3 was originally isolated from a saltmarsh [24], whereas *M. barkeri* MS was originally isolated from an anaerobic sewage digestor [31]*. M. voltae* and *M. barkeri* were provided with canonical Fe and S sources (Fe(NH_4_)_2_(SO_4_)_2_ • 6H_2_O and Na_2_S • 9H_2_O or L-cysteine HCl, respectively), laboratory synthesized nanoparticulate FeS_2_ or FeS_(mack)_, or powdered specimen FeS_2_ (63–125 µm diameter) as their sole Fe and S sources. All minerals used in our experiments were subjected to multiple washing steps to remove trace Fe and S sources that may have formed during synthesis or mineral crushing. X-ray diffraction spectra of minerals used in growth experiments confirm that they correspond to previously characterized FeS_2_ or FeS_(mack)_ standards (SOM Fig. S1). The size distribution of laboratory synthesized FeS_2_, determined via field emission scanning electron microscopy (FE-SEM), ranged from 0.22 to 4.49 µm with small framboids commonly aggregated into larger particles (SOM Fig. S2). Growth was monitored via direct cell counts (*M. voltae*) or DNA yield due to cell aggregation (*M. barkeri*), and methanogenesis activity was monitored by methane (CH_4_) production via gas chromatography.

## Materials and methods

### Preparation of synthetic pyrite

All chemicals used in mineral synthesis were American Chemical Society grade or higher. All glassware was first washed in 10% nitric acid and rinsed three times with 18.2 Ω MilliQ H_2_O (MQ H_2_O). Pyrite (FeS_2_) was synthesized in the lab according to Berner [32]. Briefly, within an anaerobic chamber (Coy Labs, Grass Lake, MI), 14.4 g Na_2_S • 9H_2_O and 16.7 g FeSO_4_ • 7H_2_O were separately dissolved in 50 ml of anoxic MQ H_2_O. These two solutions were then combined into a 500 ml bottle and stirred vigorously for 15 min, at which point 2.1 g of elemental sulfur was added. The bottle was then sealed with a butyl rubber stopper, removed from the chamber, and bubbled with N_2_ passed over heated (200 °C) and H_2_-reduced copper shavings for 45 min. Following purging, the solution was incubated anoxically for 4 days at 65 °C and then for another 4 days at 85 °C. After incubation, the FeS_2_ was washed (via centrifugation and decanting) in the following series to remove unreacted HS^−^, Fe(II), FeS, and S^0^: four rinses with 1 N HCl, one rinse with boiling 6 N HCl, two rinses with MQ H_2_O, three rinses with >99.5% acetone, and, finally, three rinses with 0.2 µm filter-sterilized MQ H_2_O. After washing, the FeS_2_ was pelleted via centrifugation, brought into an anaerobic chamber, the aqueous phase decanted, and the pellet resuspended in sterile, anoxic MQ H_2_O in a sterile serum bottle and was stoppered. Finally, the headspace of the bottle was purged with 0.2 µm filtered N_2_ gas. The % w/v of the slurry was determined by drying 1 ml of slurry in triplicate under N_2_ in pre-weighed glass serum bottles.

Mackinawite (FeS_(mack)_) was synthesized similarly to FeS_2_. Within an anaerobic chamber 1.14 g Na_2_S • 9H_2_O and 1.67 g FeSO_4_ • 7H_2_O were separately dissolved in 10 ml of anoxic MQ H_2_O. The solutions were then combined and vigorously stirred in a 50 ml glass flask for 15 min. The FeS_(mack)_ solution was washed three times with anoxic MQ H_2_O, as described above. Inside an anaerobic chamber, the final mineral pellet was resuspended in 15 ml of 0.2 µm filter-sterilized, anoxic MQ H_2_O and then transferred to a sterile serum bottle and stoppered. Finally, the headspace of the bottle was purged with 0.2 µm filtered N_2_ gas. The % w/v of the slurry was determined as described above.

Specimen FeS_2_, obtained from Zacatecas, Mexico (Ward’s Science, Rochester, NY), was crushed with an ethanol sterilized jaw crusher (Gilson, Lewis Center, OH) in a laminar flow hood. Crushed mineral was applied to a sterile sieve stack (U.S. Standard #10/2000 μm, U.S. Standard #35/500 μm, U.S. Standard #60/250 μm, U.S. Standard #120/125 μm, U.S. Standard #230/63 μm, and catch pan; all 8″ diameter) in a laminar flow hood. The sieve stack was mechanically shaken (~275 oscillations and ~150 taps per minute) for 10 min using a Ro-Tap^®^ RX-29 mechanical sieve shaker (W.S. Tyler, Mentor, OH). Following sieving, the sieve stack was returned to the laminar flow hood and the 63–125 µm fraction was collected, washed as described above for laboratory synthesized FeS_2_, and dried at room under N_2_ in a sterile serum bottle sealed with a butyl rubber stopper.

Minerals were characterized using a SCINTAG X-1 system X-ray powder diffraction (XRD) spectrometer (XRD Eigenmann GmbH, Mannheim, Germany), a JEOL JSM-6100 scanning electron microscope equipped with an energy dispersive X-ray spectrometer (JEOL USA INC., Peabody, MA), and a Zeiss SUPRA 55VP FE-SEM (Zeiss, Oberkochen, Germany). Samples for mineral characterization were prepared by drying ~10 ml of the mineral slurry or reacted mineral in a sealed serum bottle under a stream of N_2_.

### Strains and cultivation media

*Methanococcus voltae* strain A3 was obtained from the American Type Culture Collection (ATTC-BAA-1334). *M. voltae* was grown in Fe- and S-free basal medium that contained (g l^−1^): NaCl, 21.98; MgCl_2_ • 6H_2_O, 5.10; NaHCO_3_, 5.00; NH_4_Cl, 0.50; K_2_HPO_4,_ 0.14; KCl, 0.33; CaCl_2_ • 2H_2_O, 0.10 (SOM Table S2). The basal medium was amended with 0.01 g l^−1^ Fe(NH_4_)_2_(SO_4_)_2_ • 6H_2_O and 0.48 g l^−1^ Na_2_S • 9H_2_O for Fe (II)/HS^−^ grown cells. Sulfide was added from an anoxic, sterile stock solution 30 min prior to inoculation. Basal medium was amended (each 1% v/v) with trace element, vitamin, and organic solutions. The trace element solution used was based on Whitman et al. [33] and was amended to omit Fe and replace sulfate salts with chloride salts at the same molar concentrations. The trace element solution contained (g l^−1^): nitriloacetic acid, 1.500; MnCl_2_ • 4H_2_O, 0.085; CoCl_2_ • H_2_O, 0.100; ZnCl_2_, 0.047; CuCl_2_ • 2H_2_O, 0.0683; NiCl_2_ • 6H_2_O, 0.0683; Na_2_SeO_3_, 0.200; Na_2_MoO_4_ • 2H_2_O, 0.100; Na_2_WO_4_ • 2H_2_O, 0.100. The vitamin solution contained (g l^−1^): pyridodoxine HCl, 0.01; thiamine HCl, 0.005; riboflavin, 0.005 g; nicotinic acid, 0.005; calcium D(+) pantothenate, 0.005; biotin, 0.002; folic acid, 0.002; cobalamin, 0.0001. The organics solution consisted of 1 M sodium acetate • 3H_2_O, 75 mM L-leucine HCl, and 75 mM L-isoleucine HCl. Cultures of *M. voltae* grown with N_2_–CO_2_ were supplemented with a 40% (wt/v) sodium formate stock solution added to a final concentration of 0.4% (v/v) prior to inoculation.

*Methanosarcina barkeri* strain MS was purchased from the ATCC (BAA-2329) and grown on Fe- and S-free basal medium that contained (g L^−1^): NaCl, 1.0; MgCl_2_ • 6H_2_O, 0.4, NaHCO_3_, 2.00; NH_4_Cl, 0.54; KCl, 0.5; K_2_HPO_4_, 0.35; KH_2_PO_4_, 0.23; CaCl_2_ • 2H_2_O, 0.10 (SOM Table S3). The basal medium was amended with 0.01 g l^−1^ Fe(NH_4_)_2_(SO_4_)_2_ • 6H_2_O and 0.35 g l^−1^ L-cysteine HCl for Fe(II)/cysteine grown cells. Sterile, anoxic stock trace metals and vitamins solutions were prepared and added to the basal medium (1% v/v final concentration) prior to inoculation. The iron-free SL-10 trace metals solution contained (g l^−1^): ZnCl_2_, 0.070; MnCl_2_ • 4H_2_O, 0.100; H_3_BO_3_, 0.006; CoCl_2_ • 6H_2_O, 0.190; CuCl_2_ • 2H_2_O, 0.002; NiCl_2_ • 6H_2_O, 0.024; Na_2_MoO_4_ • 2H_2_O, 0.036. The vitamin solution used was as described for *M. voltae* above. Sterile, anoxic stock solutions of methanol and acetate were added to final concentrations of 0.5% (v/v) and 40 mM, respectively.

All medium components, with the exception of NaHCO_3_, were dissolved in MQ H_2_O and then boiled for 15 min. After boiling, the bottle containing the medium was sealed with a butyl rubber stopper and sparged with N_2_ passed over a heated copper column for 1 h L^−1^ of medium. After degassing, the medium was sealed, brought into an anaerobic chamber, and NaHCO_3_ was added as specified above. After adding these components, the pH of the medium was adjusted to 7.00 using anoxic 2 M HCl or 1 M NaOH. Seventy-five ml of media were dispensed into 165 ml serum bottles (DWK Life Sciences, Milville, NJ) that had been acid-washed in 10% nitric acid and sealed with black rubber stoppers (Glasgerätebau Ochs GmbH, Bovenden, Germany) and aluminum crimp caps. The serum bottles were then brought out of the chamber and the headspace was purged with N_2_ for 30 min. The bottles were then autoclaved for 20 min at 123 °C.

Synthesized FeS_2_ was added to microcosms from the prepared slurry at a final concentration of 2 mM of Fe, and specimen FeS_2_ powder was added to microcosms at 1.5 g per 75 ml of medium (167 mM). This amount was chosen to normalize for differences in the estimated surface area between laboratory synthesized FeS_2_ and powdered specimen FeS_2_. Surface area of laboratory synthesized FeS_2_ was estimated by calculating the average particle size of synthesized FeS_2_ framboids (0.74 µm diameter) then calculating surface area of the particles assuming framboids were octahedral. Briefly, ten fields of view were randomly collected using back-scattered secondary electron imaging on a Zeiss SUPRA 55VP FE-SEM. The images were imported into ImageJ software (Rasband, W.S., ImageJ, U.S. National Institutes of Health, Bethesda, Maryland, USA, https://imagej.nih.gov/ij/, 1997–2018.) and particle sizes were calculated using the “Analyze Particles” function. The surface area of specimen FeS_2_ was estimated assuming an average particle diameter of 94 µm, the midpoint of the 63–125 µm size fraction, and octahedral form.

### Cultivation procedures

Cultures of *M. voltae* and *M. barkeri* were maintained by weekly transfers (10% v/v) into fresh medium with Fe(II) as Fe source, HS^−^ or L-cysteine as S source, and formate or methanol as methanogenesis substrate, respectively. Cells were washed prior to inoculation by pelleting them in sealed 50 ml centrifuge tubes (Globe Scientific, Mahwah, NJ) at 4700 × *g* for 20 min at 4 °C in a swing-out bucket rotor. Spent medium was decanted in an anaerobic chamber and the cell pellet was resuspended in sterile and anoxic Fe/S-free basal medium. All experiments used washed *M. voltae* and *M. barkeri* cells grown with 26 µM Fe(II) and either 2 mM HS^−^ or 2 mM L-cysteine as inoculum (10% v/v), respectively. After inoculation, the headspaces of microcosms were flushed with an 80:20 (v/v) mixture of N_2_–CO_2_ gas that had been passed through a 0.2 µm filter for at least 15 min before being pressurized (final pressure of 3.14 atm). *M. voltae* and *M. barkeri* were incubated at 38 °C. All cultures were incubated statically on their sides to minimize disruption of microbe-mineral interactions while maximizing gas diffusion.

### Measurement of activity and growth

Headspace gas from microcosms was sampled with a N_2_ flushed syringe and stopcock and diluted with ultra-high purity N_2_ into CaliBond bags (Calibrated Instruments Inc., Manhasset, NY) prior to CH_4_ determination. CH_4_ was determined by gas chromatography by injecting 5 ml of sample into an injector valve set at 55 °C into an SRI 8610C gas chromatograph (SRI instruments, Torrance, CA) equipped with a 4.5 m × 0.125″ OD Hayesep DB 100/120 packed column with the oven set to 44 °C (Valco Instrument Company Inc., Houston, TX). CH_4_ was detected by a flame-ionization detector set at 156 °C with He as carrier gas. CH_4_ peak area values were converted to ppm using a standard CH_4_ curve (EGAS Depot, Nampa, ID). Dissolved HS^−^ was determined via colorimetry (670 nm) using the methylene blue assay [34] and converted to total HS^−^ (dissolved and gas phase) using Henry’s law. Absorbance was measured using a Genesys 10S Vis Spectrophotometer (Thermo Scientific, Waltham, MA).

Growth in cultures of *M. voltae* was determined by direct counting of cells using a Petroff-Hausser counting chamber on a Nikon YS100 light microscope with a ×100 oil objective (Nikon, Tokyo, Japan). Prior to counting, cells were concentrated by centrifugation at 15,000 × *g* for 15 min in a fixed-angle rotor. Despite efforts to minimize cell aggregation in cultures of *M. barkeri*, cells still aggregated necessitating an alternative approach to quantify growth. Quantification of DNA from *M. barkeri* was used as a proxy for cell growth. DNA was extracted using the FastDNA SPIN kit for soils (MP Biomedicals, Santa Ana, CA) with a modified protocol. First, 2 ml of culture were pelleted via centrifugation at 20,000 × *g* for 30 min at 20 °C. The supernatant was carefully decanted and 489 µl of sodium phosphate buffer (MP Biomedicals, Santa Ana, CA) and 61 µl of MT lysis buffer (MP Biomedicals) were used to resuspend the cell pellet. The resuspended pellet and buffer mixture were subjected to three rounds of freezing at −80 °C and thawing at 70 °C. The cell and lysis buffer mixture were transferred to a Lysis E tube (MP Biomedicals), homogenized on a bead beater for 40 s (Biospec Products, Bartlesville, OK), then centrifuged 14,000 × *g* for 15 min at 4 °C to pellet cell debris. DNA in the supernatant was quantified using a Qubit HS dsDNA assay (Invitrogen, Carlsbad, CA).

### Dialysis membrane experiments

Dialysis membranes of varying pore sizes were used to examine the requirement for direct cell contact with FeS_2_ or the size of extracellular proteins involved in catalyzing its reduction. A 12 cm length of Spectra/Por (Spectrum Labs Inc., Rancho Dominguez, CA) 3 dialysis tubing with either 3.5, 25, 50, or 100 kDa molecular weight cutoff was used for each bottle. The dialysis membranes were first washed by three cycles of soaking precut tubing in MQ H_2_O for 30 min at 65 °C, followed by decanting and rinsing with fresh MQ H_2_O. The tubing was then transferred to a bottle containing 50% ethanol, and the submerged membranes were then incubated overnight at 65 °C. The tubes were then handled in a UV-sterilized molecular hood with ethanol sterilized gloves and forceps where they were tied at one end with ethanol-soaked monofilament line (Berkley, Columbia, SC). To seal the bags in preparation for addition of FeS_2_, the end of the tubing was first folded over twice, then crimped with a dialysis clip (Spectrum Labs Inc., Rancho Dominguez, CA). The clip was then removed, leaving a fold that was amenable to tying with monofilament. The tubing was then returned to the 50% ethanol bottle, sparged with N_2_ gas for 1 h, and brought into an anaerobic chamber. Monofilament line rather than dialysis clips was used to facilitate the entry of tubing into narrow mouthed (20 mm diameter) serum bottles for cultivation experiments.

Inside the anaerobic chamber, the dialysis bags were rinsed in sterile and anoxic MQ H_2_O to remove residual ethanol. Then, laboratory -synthesized FeS_2_ was added to dialysis bags as a slurry (2 mM final concentration Fe once in medium bottles), and the bags were resealed as described above with monofilament line. Finally, the outsides of all bags were rinsed with anoxic and sterile MQ H_2_O and were transferred to pre-sterilized 165 ml serum bottles containing 75 ml of basal medium amended with vitamins, trace metals, and organics solution. The bottles were sealed with sterilized butyl rubber stoppers and purged with an 80:20 mixture of 0.2 µm filtered N_2_:CO_2_ for 45 min to remove gas originating from the anaerobic chamber and were then pressurized (final pressure of 3.14 atm). The bottles were then inoculated with washed, Fe(II)/HS^−^-grown cells (10% v/v inoculum). Positive controls contained 3.5 kDa dialysis membranes and FeS_2_ that was not enclosed in the membrane. Unamended controls contained 3.5 kDa dialysis membranes without any added Fe or S source.

### Electron microscopy

Subsamples of cultures of *M. voltae* for use in FE-SEM imaging analyses were collected and incubated in 10 ml of freshly prepared 2% glutaraldehyde solution in 0.1 M phosphate buffer for 2 h at room temperature. Glutaraldehyde-fixed samples were applied to a Au-sputtered 0.2 µm black Isopore polycarbonate filter (MilliporeSigma, Burlington, MA). The filter was subjected to an ethanol series (25, 50, 70, 85, 95, and 100%) for complete dehydration, then stored dry at 4 °C until imaging in the Imaging and Chemical Analysis Laboratory at Montana State University. Images were taken using a high-resolution FE-SEM (Supra 55VP, Zeiss, Thornwood, NY) with a primary electron beam energy of 1 keV at different magnifications. Samples were mounted on the FE-SEM holder using double-sided carbon tape and sputtered with a thin film of iridium for conductivity before loading to the FE-SEM chamber. Elemental mapping was acquired using energy dispersive X-ray spectroscopy with a Scanning Auger Electron Nanoprobe-Physical Electronics 710 and 10 and 20 kV energy beam.

## Results and discussion

### Reductive dissolution of FeS_2_ at low temperature

**Fig. 1:**
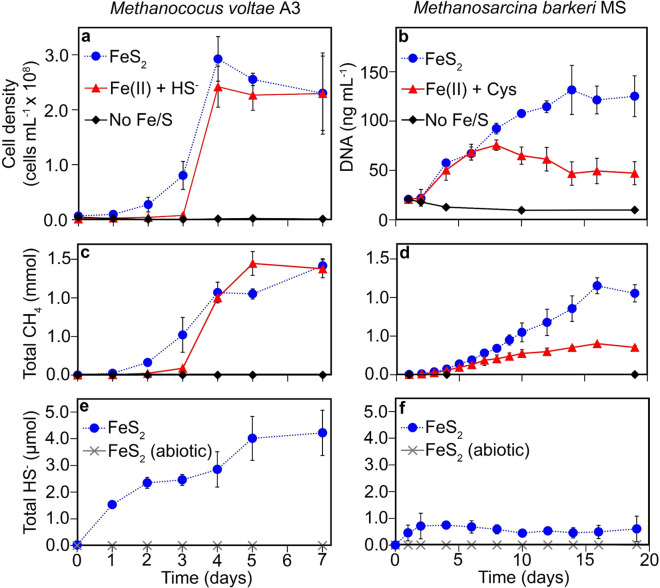
Growth and activity of methanogens on different iron and sulfur sources. Production of biomass, methane, and sulfide in cultures of *Methanococcus voltae* provided with formate (**a**, **c**, **e**, respectively) or *Methanosarcina barkeri* provided with methanol and acetate (**c**, **d**, **f**, respectively) as the methanogenesis substrate when grown with different iron and sulfur sources in defined medium. Legends in **a** and **c** and in **b** and **d** are the same. Ferrous iron and sulfide or cysteine were added to final concentrations of 26 µM and 2 mM, respectively. Laboratory synthesized pyrite was added to a final concentration of 2 mM Fe. CH_4_, total methane; HS^−^, total sulfide; Fe(II), ferrous iron; Cys, cysteine; FeS_2_, pyrite. Data shown are the mean values for each condition with error bars reflecting the standard deviation of three replicate biological reactors; a single reactor for abiotic controls was monitored and thus error bars are not presented. Total HS^−^ was monitored only in the FeS_2_ growth conditions. See Extended Data File 1 for CH_4_ and HS^−^ data.

Cultivation of *M. voltae* and *M. barkeri*, when provided with formate or methanol and acetate as the methanogenesis substrates, respectively, resulted in significant cell and CH_4_ production in cultures containing nanoparticulate FeS_2_ or canonical forms of Fe and S but were not observed in cultures unamended with Fe or S (Fig. 1a–d and Extended Data File 1). Growth of both *M. voltae* and *M. barkeri* was also observed with powdered specimen FeS_2_ as the sole Fe and S source (SOM Fig. S3). These observations indicate that both synthetic nanoparticulate FeS_2_ and specimen FeS_2_, the latter of which should be reflective of FeS_2_ commonly encountered by methanogens in natural environments, can serve as the sole Fe and S source for these cells.

Sulfide (referred to as HS^−^ from here on) was detected in cultures of *M. voltae* and *M. barkeri* provided with nanoparticulate FeS_2_ but not in abiotic controls; concentrations of HS^−^ increased with increased production of biomass and CH_4_ (Fig. 1e, f and Supplementary Data File 1). This is consistent with cells catalyzing the reductive dissolution of FeS_2_ to produce HS^−^. The accumulation of HS^−^ suggests that the rate of FeS_2_ reduction by methanogens exceeds the rate of cellular S uptake. To the extent that cells are not taking up HS^−^ (discussed below), the amount of HS^−^ that accumulated in the growth medium can be combined with the predicted cellular S requirement per cell to provide a conservative estimate of the amount of FeS_2_ reduction in laboratory grown cultures. The estimated S content of log phase *Methanococcus maripaludis*, a close relative of *M. voltae*, is 349 nmol per mg protein [22]. The amount of protein in log phase *M. voltae* is 8.88 × 10^−10^ mg cell^−1^ (E.S. Boyd, unpublished data). As such, an individual *M. voltae* cell during log phase is estimated to contain 0.31 fmol S. When this is combined with the amount of HS^-^ that accumulates during FeS_2_ reduction, as normalized to an individual cell (0.15 fmol cell^−1^, see Extended Data File 1), it is conservatively estimated that *M. voltae* catalyzes the reduction of 0.23 fmol FeS_2_ cell^−1^ during log phase growth.

Importantly, acid labile Fe(II) was not detected in spent medium via the ferrozine assay (~11 µM lower detection limit) at any point during the growth of *M. voltae* or *M. barkeri* (data not shown). This could indicate that the cells maintain aqueous Fe(II) below the limits of detection of our assay or that the Fe(II) phase that forms via FeS_2_ reduction (reverse of Eqs. (2)–(4)) undergoes rapid precipitation on the mineral surface. A coupled reductive dissolution and precipitation reaction has been proposed for abiotic reduction of FeS_2_ at high temperature, with iron monosulfide as mackinawite or pyrrhotite forming as the predominant Fe(II) phase(s) on the surface of FeS_2_ [10]. Together, these data indicate that representatives of both Class I and II methanogens can catalyze the reduction of FeS_2_ at low temperatures (≤38 °C) and raise the question of the form(s) of Fe and S that is assimilated to meet biosynthetic demands.

**Fig. 2:**
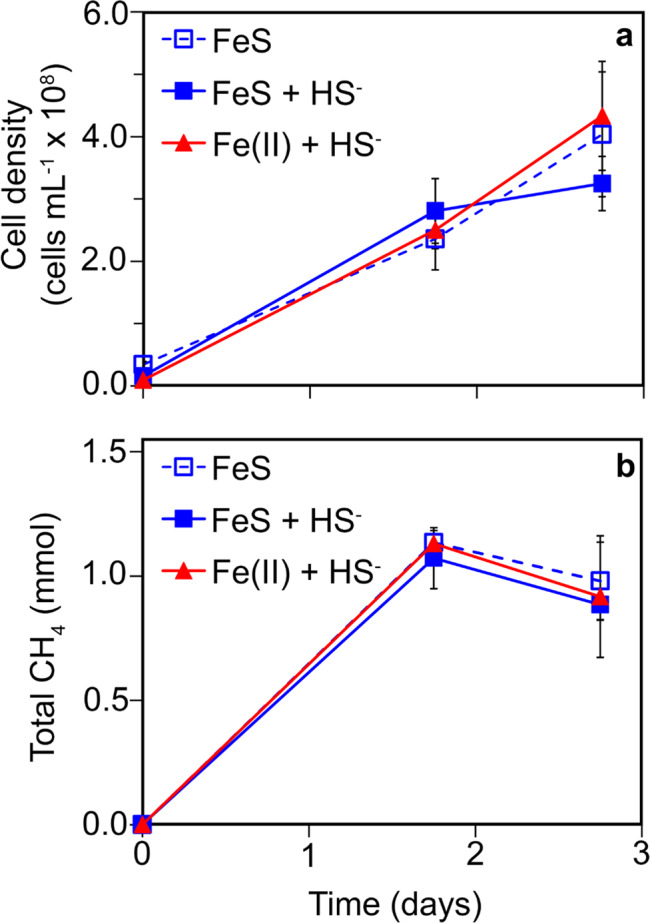
Growth and activity of *Methanococcus voltae* provided with mackinawite as the sole iron and sulfur source. Production of cells (**a**) and methane (**b**) for *M. voltae* cultures. Cells were grown with synthetic mackinawite (FeS) added to a final concentration of 2 mM Fe with or without added 1 mM added HS^−^. Cells provided with Fe(II) and HS^−^ served as the positive control. CH_4_, methane; Fe(II), ferrous iron; HS^−^, sulfide; FeS, mackinawite. Data shown are the mean values for each condition with error bars reflecting the standard deviation of three replicate biological reactors. See Extended Data File 1 for CH_4_ and HS^−^ data.

One source of Fe and S that could potentially support growth of methanogens during the reductive dissolution of FeS_2_ is FeS_(mack)_, which would be expected to be produced if biological FeS_2_ reduction is taking place via the reverse reaction of Eqs. (3) or (4). Further, FeS_(mack)_ is a commonly detected form of iron monosulfide in low temperature settings [35] and is a possible precursor to the formation of FeS_2_ in these environments [29, 36]. Equilibrium dissolution of FeS_(mack)_ in non-acidic (pH > 6.0) aqueous solutions results in the formation of FeS_(aq)_ as the dominant form of Fe(II) (reverse of Eq. (2)) when HS^–^ is at a concentration exceeding 1 µM [28]. Thus, to further evaluate the potential form of Fe and S supporting growth of cells, *M. voltae* was provided with nanoparticulate, laboratory -synthesized FeS_2_ or FeS_(mack)_ in the presence or absence of 1 mM added HS^–^. This concentration of HS^−^ was chosen such that any aqueous Fe(II) either dissociated from FeS_(mack)_ or from a trace laboratory contaminant, if present, would predominantly be in the form of FeS_(aq)_ [28, 29]. *M. voltae* cultures showed no apparent difference in growth or activity with FeS_(mack)_ in the presence or absence of added HS^−^ and these did not differ from the Fe(II)/HS^−^ positive control (Fig. 2). *M. barkeri* also demonstrated growth on FeS_(mack)_ as the sole Fe and S source (SOM Fig. S4). We also tested the growth of *M. voltae* on FeS_2_ in the presence or absence of 1 mM HS^−^ and observed growth and surprisingly also observed HS^−^ production from FeS_2_ even in the presence of added HS^−^ (SOM Fig. S5). Sulfide concentrations could not be accurately monitored in growth experiments with FeS_(mack)_ given that this mineral is partially soluble, acid labile, and forms nanoparticles (~2 nm in size) [29] that cannot be readily size fractionated; these features interfere with the sulfuric acid-based methylene blue assay that was used to quantify HS^−^. Collectively, these results suggest that cells can use mineral forms of Fe and S (as FeS_2_ and FeS_(mack)_) to meet both Fe and S demands and that they may even prefer these forms over canonical forms (e.g., Fe(II) or HS^−^/cysteine).

### Comparing growth on FeS_2_ to growth under Fe or S limiting conditions

To determine whether *M. voltae* can directly assimilate aqueous HS^-^ and to verify that trace Fe or S in the cultivation medium was not sufficient to meet cellular demands, we compared the growth and activity of *M. voltae* cultures provided with only FeS_2_ to those provided with only Fe(II) or only HS^−^ (SOM Fig. S6). When cells were provided with only Fe(II), there was no production of cells and CH_4_ production was minimal. However, production of cells and CH_4_ was observed in cultures provided with only HS^−^, albeit at 40% of the cell yield for FeS_2_-grown cells. These results indicate that trace Fe in the medium can partially fulfill biological demands in cultures provided with HS^−^ but that trace S cannot, consistent with a higher demand for S than Fe in cells [22]. Our findings agree with the apparent ability of Class I and Class II methanogens to assimilate HS^–^ during growth [22, 37]. However, given that HS^−^ readily reacts with Fe(II) to form FeS_(aq)_ and FeS_(mack)_ and the fact that both HS^−^ and Fe(II) tend to be provided in the growth medium of methanogens, it is likely that these and other Class I and II methanogens can assimilate FeS_(aq)_ or iron monosulfide precipitates and may supplement S demands by assimilating free HS^−^.

### Interactions between methanogens and FeS_2_

**Fig. 3:**
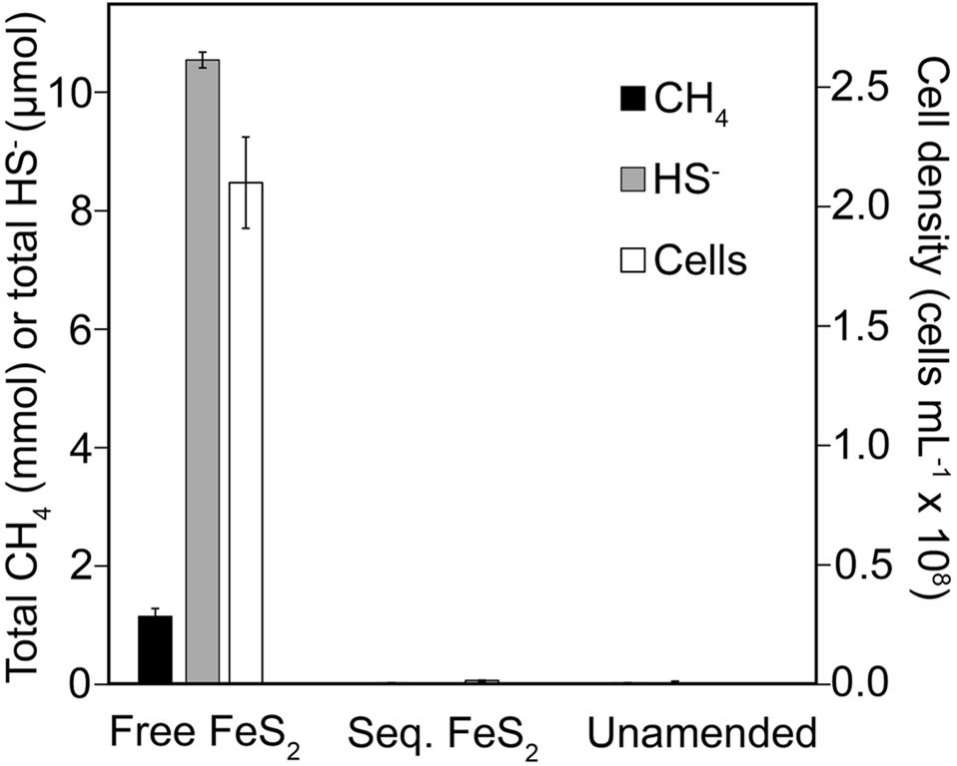
Production of cells, methane, and sulfide in cultures of *Methanococcus voltae* provided with formate as the methanogenesis substrate and pyrite (FeS_2_) in solution (free FeS_2_), FeS_2_ sequestered in dialysis membranes (seq. FeS_2_), or without FeS_2_ or other sources of Fe or S (unamended). FeS_2_ was added to a final concentration of 2 mM Fe. The cell density was determined after 5 days. Data shown are the mean values for each condition with error bars reflecting the standard deviation of three replicate biological reactors. CH_4_, methane; HS^−^, sulfide. See Supplementary Table 3 for data used to generate the plot.

The requirement for cells to associate with the surface of FeS_2_ for mineral-dependent growth was investigated using dialysis membrane experiments. *M. voltae* was grown with formate as the electron donor and with FeS_2_ free in solution or sequestered in dialysis membranes with 3.5 kDa pore size to limit direct access to the mineral surface. Growth of *M. voltae* and the reduction of FeS_2_ was assessed by monitoring production of cells, CH_4_, and HS^−^ (Fig. 3 and SOM Table S3). Cells, CH_4_, and HS^−^ were produced when FeS_2_ was provided free in solution but not when FeS_2_ was sequestered in dialysis tubing with a 3.5 kDa pore size (Fig. 3). Cells, CH_4_, and HS^−^ were also not detected when FeS_2_ was sequestered in dialysis tubing with 25, 50, or 100 kDa pore sizes (data not shown), which would allow for the passage of small extracellular proteins or metabolites across the dialysis membrane that may be involved in FeS_2_ reduction or acquisition of reductive dissolution products. Collectively, these results indicate that cells require direct contact with FeS_2_ to catalyze its reduction and/or that cells require direct access to FeS_2_ to acquire Fe and S following the coupled reductive dissolution-precipitation reaction. Alternatively, these results could point to high molecular weight (>100 kDa) extracellular proteins, metabolites, or other cellular assemblages (such as enzyme containing vesicles) as being involved in catalyzing FeS_2_ reduction or in acquiring mineral-associated Fe and S. The involvement of vesicles in mineral sulfide oxidation has been demonstrated in the crenarchaeote *Metallosphaera sedula*, where they were shown to adhere to and promote the oxidation and solubilization of chalcopyrite (CuFeS_2_) [38].

**Fig. 4:**
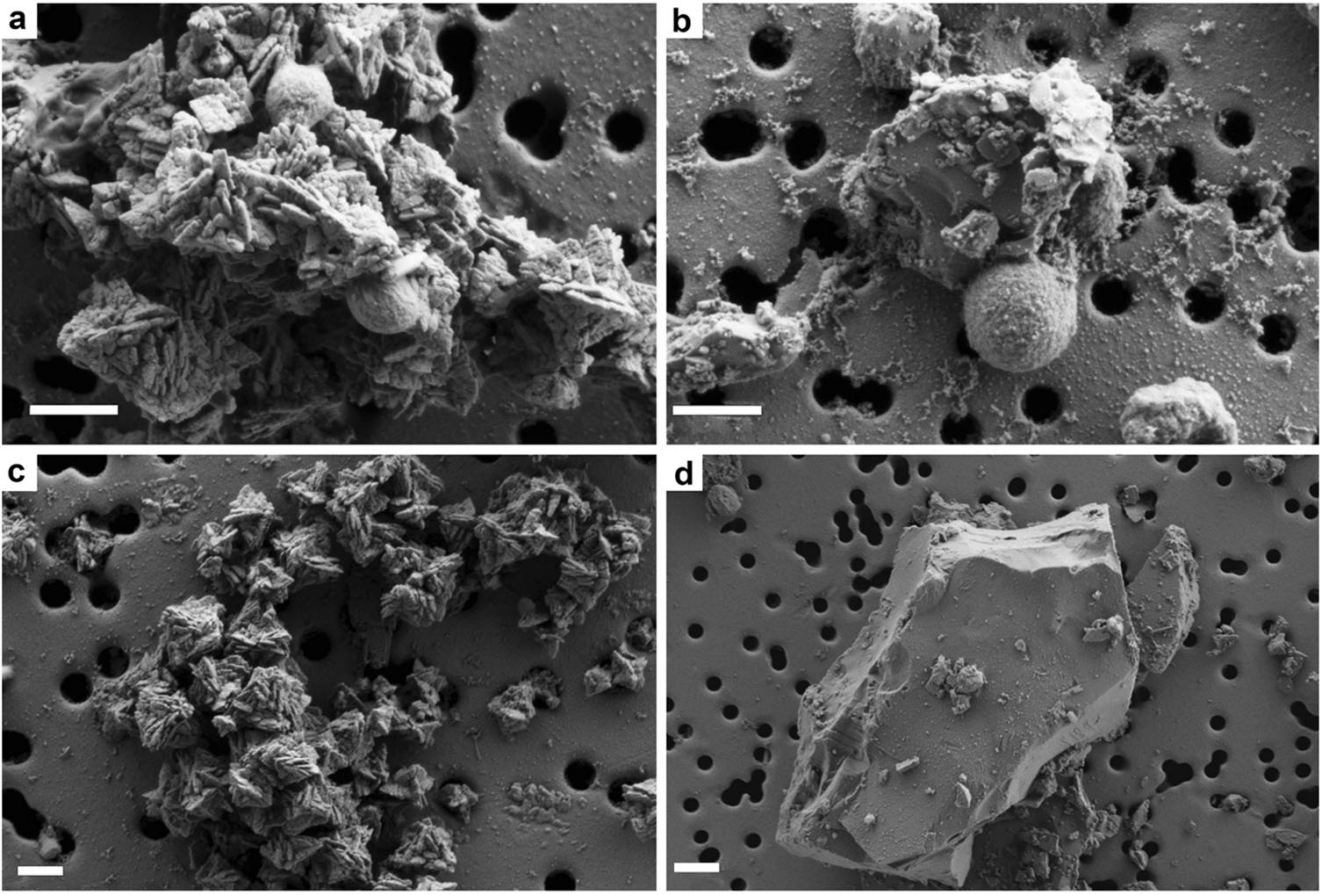
Field emission scanning electron micrographs (FE-SEM) of *Methanococcus voltae* cells grown with synthetic and specimen pyrite as the sole Fe and S source. **a** Cells of *M. voltae* grown with synthetic pyrite (FeS_2_) or **b** specimen FeS_2_. **c** Synthetic FeS_2_ or **d** specimen FeS_2_ in the absence of *M. voltae*. Samples were fixed with 2% glutaraldehyde before FE-SEM imaging on gold-sputtered 0.2 µm polycarbonate filters. Scale bar equals 0.5 µm in **a**–**c** and 1.0 µm in **d**.

We therefore examined associations between *M. voltae* (and possible vesicles) and both synthetic and specimen forms of FeS_2_ using FE-SEM imaging (Fig. 4). We did not observe any vesicles, but this is potentially attributable to the 0.4 µm pore size used for the EM grid which may not have allowed vesicles to be visualized. Rather, formate-grown *M. voltae* cells provided with FeS_2_ were observed to be attached to the mineral during growth, perhaps mediated by extracellular appendages (Fig. 4a, b and SOM Figs. S7 and S8). Archaeal flagella, or archaella, are well characterized in *M. voltae* and other Archaea where they function in motility, attachment to surfaces, and in biofilm formation [39]. The apparent affinity for cells to attach to the mineral surfaces and form biofilms is further supported by observations that when cells are grown with FeS_2_, CH_4_ production (seen as bubble generation) and cellular biomass (biofilms) are co-localized with settled synthetic (Supplementary Video 1) or specimen (Supplementary Video 2) mineral grains. While one would expect bubble generation to occur more extensively on nucleation points under highly saturated conditions, this phenomenon is also observed early in growth when CH_4_ levels are low. These observations provide further evidence indicating that methanogens likely attach to mineral sources to meet Fe and S biosynthetic demands through bio-catalyzed reductive dissolution.

## Conclusions

Data presented here reveal that representatives of both Class I and II methanogens, including strains from both freshwater (*M. barkeri*) and marine (*M. voltae*) environments, can catalyze the reductive dissolution of FeS_2_. This activity results in the liberation of Fe and S, likely as precipitated secondary minerals such as FeS_(mack)_ and/or pyrrhotite, that are used to meet biosynthetic demands. These observations raise intriguing questions related to the mechanism(s) of biological FeS_2_ reduction, the mechanism(s) of iron monosulfide acquisition and assimilation, and intracellular processing and trafficking of iron monosulfide for [Fe-S] cluster, amino acid, cofactor, and vitamin biosynthesis. Further, these observations highlight the ability of methanogens to transform Earth abundant and inert iron sulfide minerals, such as FeS_2_, into co-factors for enzymes capable of interconverting substrates (e.g., CO_2_/CH_4_ and H^+^/H_2_) of ecological and biotechnological relevance. These enzymes include simple and complex [Fe-S] clusters associated with [NiFe]-hydrogenases, carbon monoxide dehydrogenases, and numerous other Fe-S metalloproteins and co-factors synthesized by methanogens. The ability of anaerobic methanogens to acquire Fe and S from abundant mineral sources long considered to be non-bioavailable necessitates a re-evaluation of the Fe and S cycles in anoxic ecosystems of today and in the geological past.

## Supplementary information

Supplemental Figures S1-S8 and Tables S1-S3

Data Set 1

Supplementary video 1

Supplementary video 2
