# Supplementary material for: Reductive dissolution of pyrite by methanogenic archaea
Source: ISME J. 2021 Jun 10;15(12):3498–507. doi: 10.1038/s41396-021-01028-3 (PMC8630215; doi:10.1038/s41396-021-01028-3)
Supplement: Supplementary file 1 — Supplemental Figures S1-S8 and Tables S1-S3 [file 41396_2021_1028_MOESM1_ESM.pdf]

SUPPLEMENTAL ONLINE MATERIALS

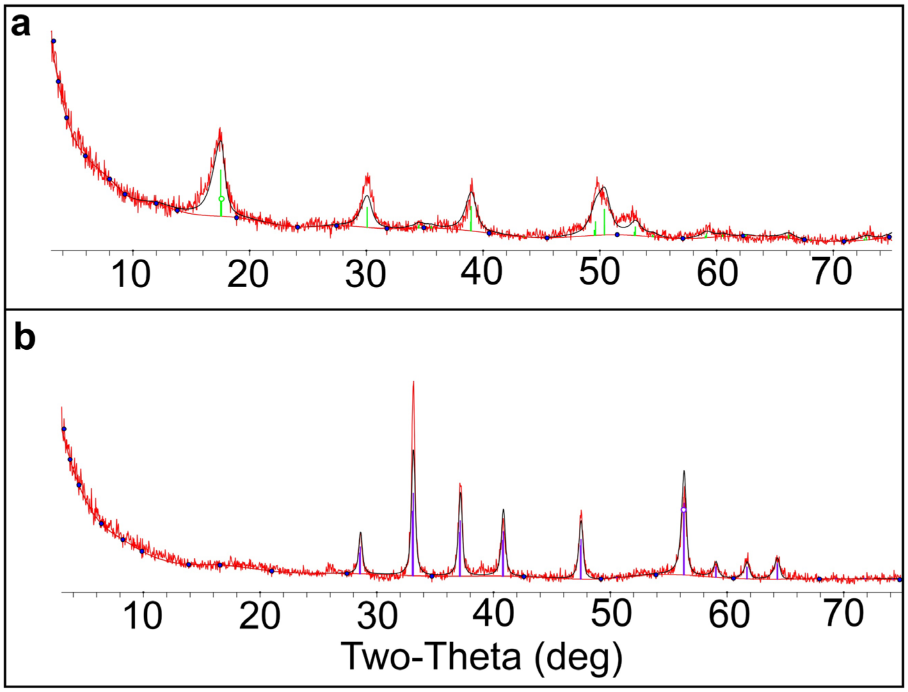

Supplementary Fig. 1. X-ray diffraction (XRD) spectra of laboratory synthesized iron monosulfide (FeS) (a) and pyrite (FeS_2) (b). Green lines in panel **a** depict published FeS references peaks [1]. Purple lines in panel **b** depict FeS_2 reference peaks [2].

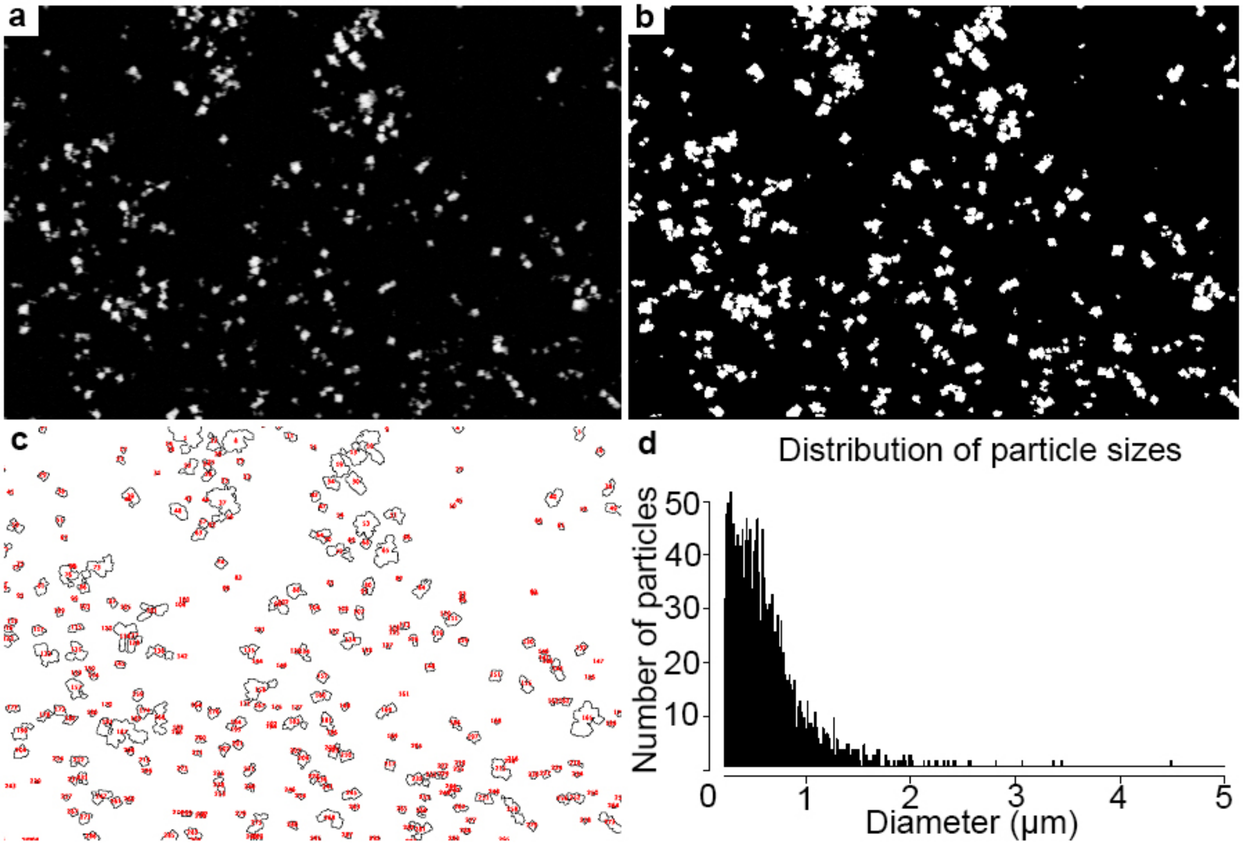

Supplementary Fig. 2. Size distribution of synthetic pyrite (FeS_2) as determined by field emission scanning electron microscopy. An aliquot of the synthesized FeS_2 was filtered onto a gold-sputtered 0.2 μm black polycarbonate filter. Images were collected using back-scattered electron imaging of mineral particles **(a)**. Images were imported into ImageJ software and threshold was adjusted to delineate particle boundaries **(b)** and then used for particle analysis **(c)**. Ten fields of view taken at 10,000 x magnification were used to determine the average particle distribution in **(d)**.

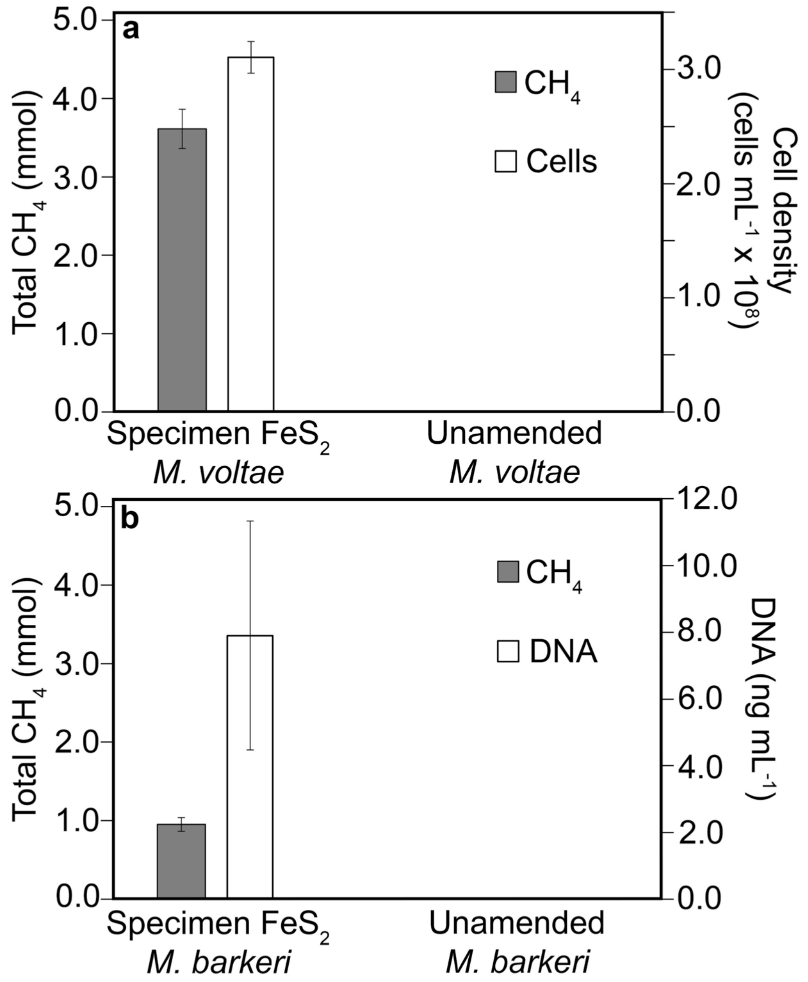

Supplementary Fig. 3. Production of total methane and biomass in cultures of *Methanococcus voltae* (A) or *Methanosarcina barkeri* (B) grown with powdered specimen pyrite (FeS_2). 1.5 g of specimen FeS_2 was added to reactors of *M. voltae* growing with formate (A) or *M. barkeri* growing with methanol and acetate (B**).** Samples for cells or DNA and methane (CH_4) were collected on day five of incubation for *M.* voltae and day 27 for *M. barkeri* when cultures were in late log phase. The unamended controls contained no added Fe and S. Data shown are the mean values for each condition with error bars reflecting the standard deviation of the mean of three replicate biological cultures.

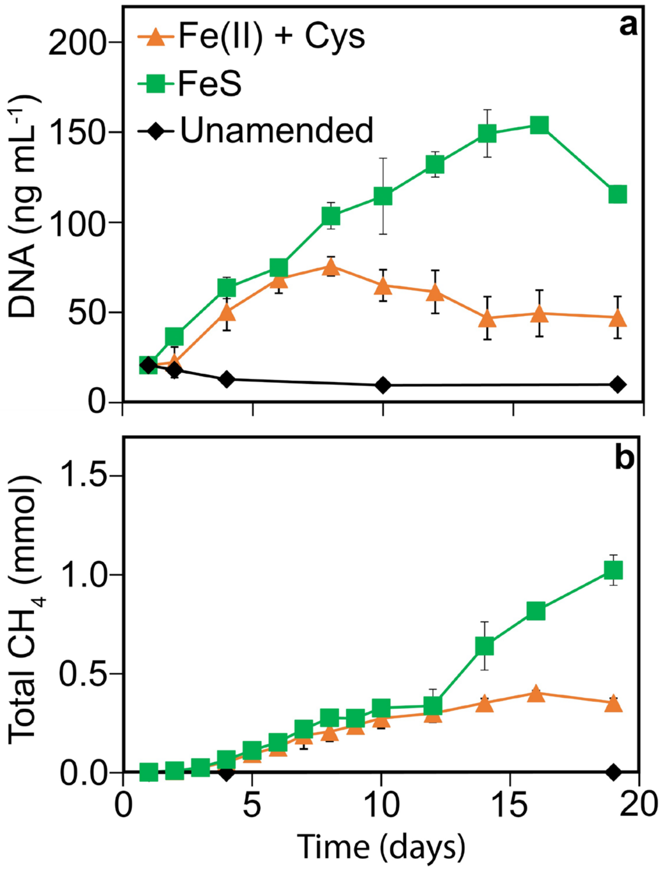

Supplementary Fig. 4. Production of cells (a) or methane (b) in cultures of *Methanosarcina barkeri* provided with methanol and acetate as methanogenesis substrates when grown with mackinawite. *M. barkeri* was grown in defined medium with mackinawite (FeS, 2 mM Fe), or with 26 µM Fe(II) and 2 mM L-cysteine, or unamended. Abbreviations: CH_4, methane; Fe (II), ferrous iron; cys, L-cysteine; FeS, mackinawite. Data shown are the mean values for each condition with error bars reflecting the standard deviation of three replicate biological reactors.

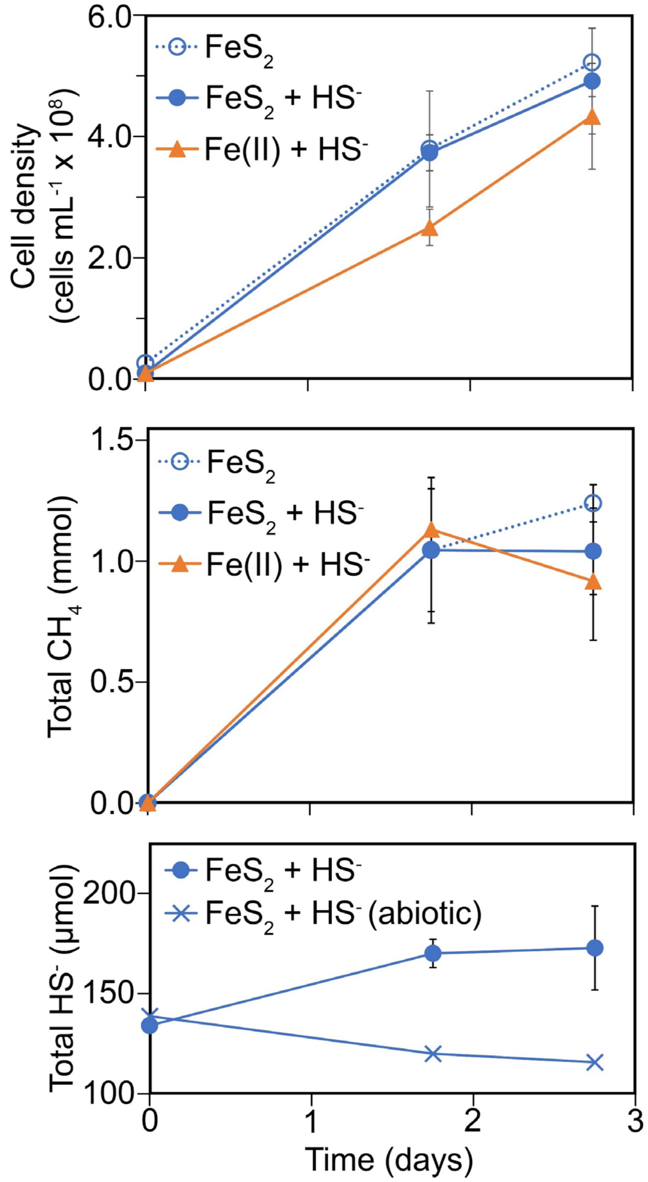

Supplementary Fig. 5. Growth of *Methanococcus voltae* with pyrite in the presence of added sulfide. *M. voltae* was grown with formate and FeS_2 added to 2 mM Fe with or without 1 mM added HS^-. Cells grown with Fe(II) and HS^- served as the positive control. Cells (**a**) and CH_4 (**b**) production were monitored in all growth conditions. HS^- production (**c**) was only monitored in FeS_2 growth conditions and an abiotic control. Abbreviations: CH_4, methane; Fe (II), ferrous iron; HS^-, sulfide; FeS_2, pyrite. Data shown are the mean values for each condition with error bars reflecting the standard deviation of three replicate biological reactors; a single reactor for abiotic controls was monitored and thus error bars are not presented.

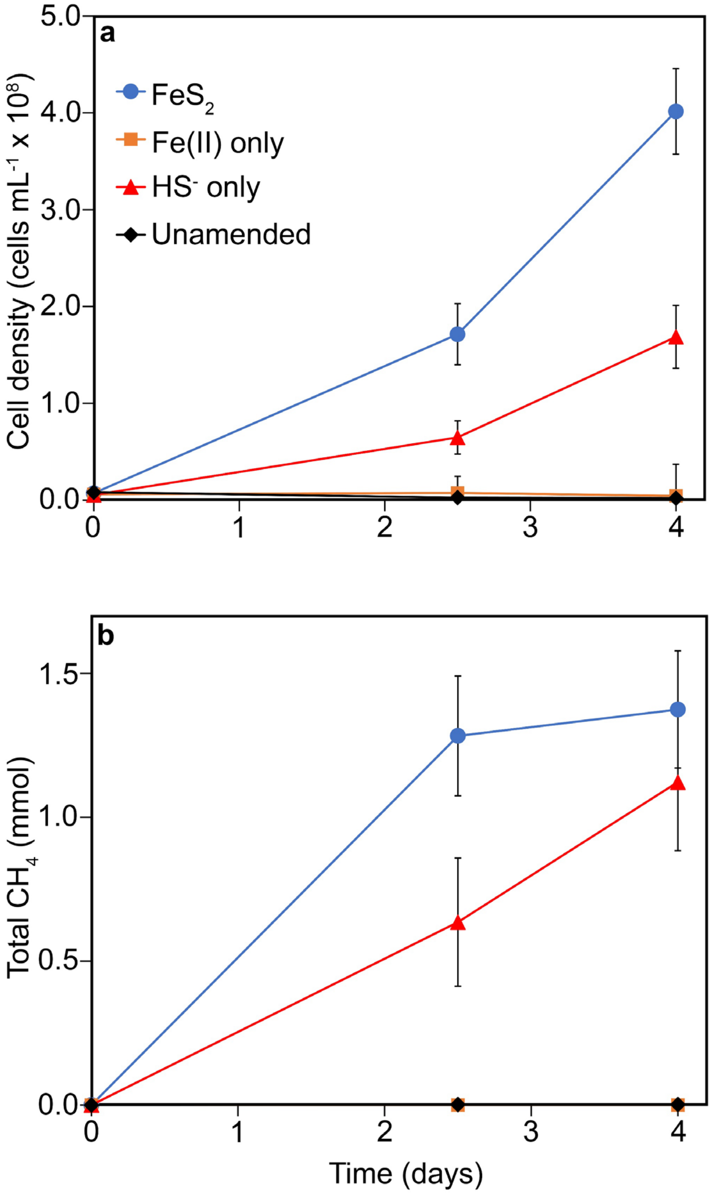

Supplementary Fig. 6. Production of cells (a) or methane (b) in cultures of *Methanococcus voltae* provided with formate as methanogenesis substrate when grown with pyrite versus iron or sulfur limiting conditions. *M. voltae* was grown in defined medium with pyrite (FeS_2, 2 mM Fe), or under Fe limiting conditions (HS^- only, 2 mM), S limiting conditions (Fe (II) only, 26 µM), or limited for both Fe and S (unamended). Abbreviations: CH_4, methane; Fe (II), ferrous iron; HS^-, sulfide; FeS_2, pyrite. Data shown are the mean values for each condition with error bars reflecting the standard deviation of three replicate biological reactors.

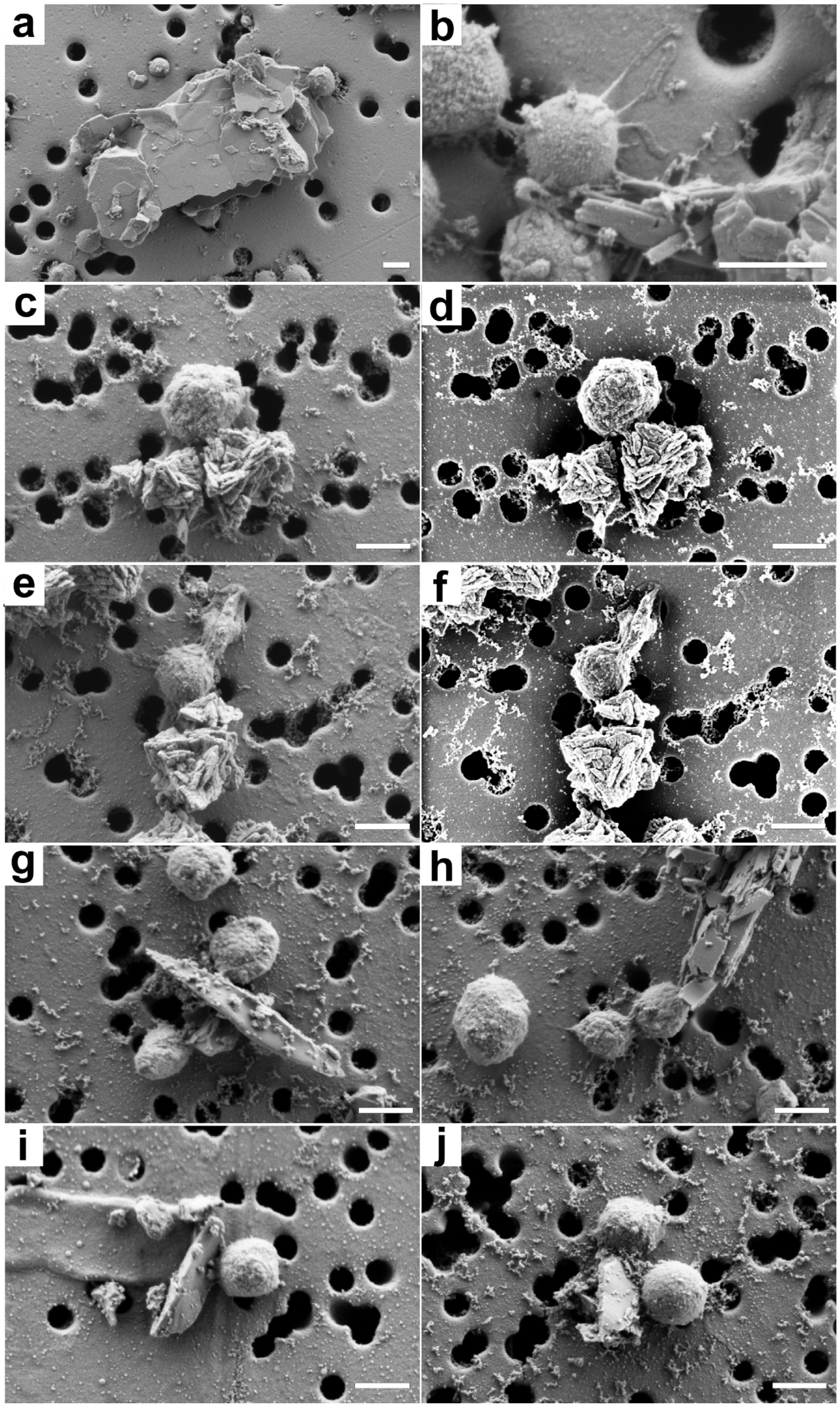

Supplementary Fig. 7. Additional field emission scanning electron micrographs of *Methanococcus voltae* and cells grown with synthetic and specimen pyrite as the sole Fe and S source. *M. voltae* cells grown with synthetic (**a-f**) and specimen (**g-j**) pyrite. Images in (**d**) and (**f**) were collected using an in-lens detector to enhance visualization of surface structure. Scale bars equal 0.5 μm in all images.

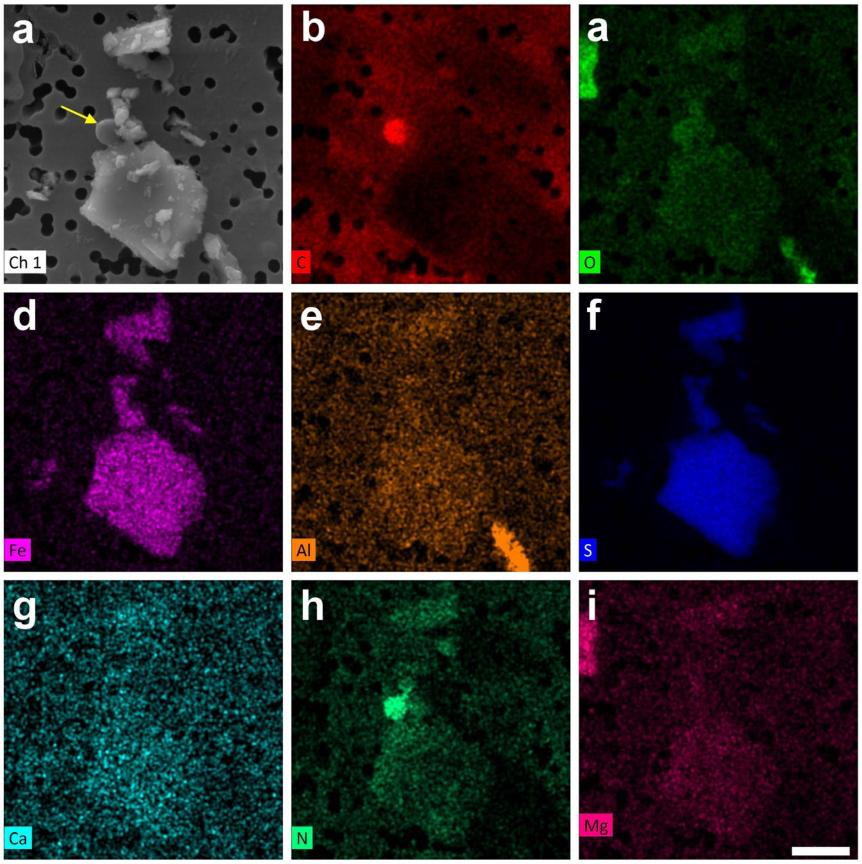

Supplementary Fig. 8. Elemental mapping of a single *Methanococcus voltae* cell attached to a grain of specimen pyrite. (**a**) Scanning electron microscopy and (**b**) Auger electron spectroscopy element maps for carbon, (**c**) oxygen, (**d**) iron, (**e**) aluminum, (**f**) sulfur, (**g**) calcium, (**h**) nitrogen, and (**i**) magnesium of *M. voltae* attached to a grain of specimen pyrite. Color intensity represents element signal with brighter being more concentrated. A single *M. voltae* cell is identified with a yellow arrow in (**a**). Scale is consistent across all panels and scale bar in (**i**) represents 1 μm.

Supplementary Table 1. Components of basal medium (g L^{-1}) and amendments used to cultivate *Methanococcus voltae.*

K_2HPO_4	0.14
KCl	0.33
$MgCl_2 \cdot 6H2O$	5.10
NH_4Cl	0.50
$CaCl_2 \cdot 2H_2O$	0.10
NaCl	21.98
$NaHCO_3$*	5.00
Organics solution**	10.00 mL
Vitamins solution**	10.00 mL
Trace element solution**	10.00 mL

* Added inside an anaerobic chamber

** *See* main text materials and methods for recipes

Supplementary Table 2. Components of basal medium (g L^{-1}) and amendments used to cultivate *Methanosarcina barkeri*

K_2HPO_4	0.35
KH_2PO_4	0.23
KCl	0.76
$MgCl_2 \cdot 6H2O$	10.17
NH_4Cl	0.50
$CaCl_2 \cdot 2H_2O$	0.10
NaCl	29.22
$NaHCO_3$*	1.00
Sodium acetate · $3H_2O$	6.80
Methanol	0.5% (v/v)
Vitamins solution**	10.00 mL
Iron-free SL-10**	10.00 mL

* Added inside an anaerobic chamber

** *See* main text materials and methods for recipes

Supplementary Table 3. Cell, methane, and sulfide production data[a] from dialysis (3.5 kDa) experiments with *Methanococcus voltae* (*See* **Supplementary Fig. 3**.)

	FeS_2 (abiotic)	FeS_2	FeS_2 in bag (abiotic)	FeS_2 in bag	Unamended
Initial cells mL^{-1}	0	1.02E+7 ± 1.38E+6	0	1.15E+7 ± 8.18E+5	1.23E+7 ± 1.31E+6
Final Cells mL^{1-}	0	2.10E+8 ± 1.10E+7	0	1.58E+6 ±1.25E+5	1.59E+6 ± 1.67E+5
Initial CH_4 (mmol)	0	0 ± 0	0	0 ± 0	0 ± 0
Final CH_4 (mmol)	0	1.15 ± 0.13	0	0 ± 0	0 ± 0
Initial HS^- (µmol)	B.D.	B.D.	B.D.	B.D.	B.D.
Final HS^- (µmol)	B.D.	10.55 ± 0.13	B.D.	B.D.	B.D.

[a]All data presented as means ± standard deviations of three biological replicates. Single reactors were used for each abiotic control and thus do not have standard deviations. *M. voltae* was grown with formate at 38 °C for five days. Abbreviations: FeS_2, pyrite; CH_4, total methane; HS^-, total sulfide; B.D., below detection.

REFERENCES

1. Bertaut EF, Burlet P, Chappert J. Sur l'absence d'ordre magnetique dans la forme quadratique de FeS. Solid State Communications. 1965; 3: 335-38.
2. Franz ED. Roentgenographische Daten der Mischphasen im System Pyrit (Fe S2) - Ferrosilit (Fe Se2) / Pyrrhotin (Fe S) - Achavalit (Fe Se). Neues Jahrbuch fuer Mineralogie. 1972; 1972: 276-80.
